# Effects of Waste Glass Powder on Rheological and Mechanical Properties of Calcium Carbide Residue Alkali-Activated Composite Cementitious Materials System

**DOI:** 10.3390/ma16093590

**Published:** 2023-05-08

**Authors:** Youzhi Chen, Xiuqi Wu, Weisong Yin, Shichang Tang, Ge Yan

**Affiliations:** 1 State Key Laboratory of Silicate Materials for Architecture, Wuhan University of Technology, Wuhan 430070, China; cyzly@whut.edu.cn (Y.C.); 303879@whut.edu.cn (X.W.); 317548@whut.edu.cn (S.T.); 2 Key Laboratory of Roadway Bridge & Structure Engineering, Wuhan University of Technology, Wuhan 430070, China; 3 Wuhan Hanyang Municipal Construction Group Co., Ltd., Wuhan 430050, China; yange1989@whut.edu.cn

**Keywords:** glass powder, fly ash, calcium carbide residue, alkali-activated materials, rheological behavior, compressive strength

## Abstract

As a municipal solid waste, waste glass undergoes pozzolanic activity when ground to a certain fineness. In this paper, calcium carbide residue (CCR) and Na_2_CO_3_ were used as composite alkali activators for a glass powder-based composite cementitious system. A total of 60% fly ash (FA) and 40% ground granulated blast furnace slag (GGBS) were used as the reference group of the composite cementitious material system, and the effects of 5%, 10%, 15%, and 20% glass powder (GP) replacing FA on the rheological behavior, mechanical properties, and microstructure of alkali-activated composite cementitious systems were investigated. The results showed that with the increase in GP replacing FA, the fluidity of the alkali-activated materials gradually decreased, the shear stress and the equivalent plastic viscosity both showed an increasing trend, and the paste gradually changed from shear thinning to shear thickening. Compared with the reference sample, the fluidity of the alkali-activated material paste with a 20% GP replacement of FA was reduced by 15.3%, the yield shear stress was increased by 49.6%, and the equivalent plastic viscosity was elevated by 32.1%. For the 28d alkali-activated material pastes, the compressive strength and flexural strength were increased by 13% and 20.3%, respectively. The microstructure analysis showed the substitution of FA by GP promoted the alkali-activated reaction to a certain extent, and more C-A-S-H gel was formed.

## 1. Introduction

In order to address global climate change and promote the reduction in CO_2_ emissions, China has proposed to reach the peak of carbon emissions before 2030 and achieve carbon neutrality by 2060, which has become one of the important development strategies for China [1,2]. Among them, the awareness of sustainable development in the construction industry has increased, and various environmentally friendly materials have been adopted as alternatives to traditional materials [3,4,5]. Using industrial solid waste as precursors and mixing them with alkaline solutions to form clinker-free alkali activated materials has been an effective method to achieve the “dual carbon” goal [6,7]. However, measures such as the optimization of the industrial structure of thermal power plants and the use of clean energy for power generation have led to a decrease in FA production and a continuous increase in the price of FA, thus increasing the cost of new building materials to a certain extent [8,9].

At present, alkali-activated materials with GGBS, FA, and their mixtures as precursors have been most intensively studied, usually mixed with certain concentrations of alkaline solution, and their various properties have been extensively investigated [10,11,12]. Ahmed et al. [13] used 5% Na_2_CO_3_ to activate the FA–GGBS system. It was concluded that Na_2_CO_3_ as an activator was easier to mix compared to sodium hydroxide, and the mechanical properties of the hardened specimens improved significantly with the increase in the Na_2_CO_3_ dosage. Li et al. [14] systematically investigated Na_2_CO_3_ used as a single activator to activate GGBS systems and found that the induction period of GGBS hydration could be shortened and the production of C-(N)-A-S-H could be promoted. However, strong alkaline activators were corrosive and could not be premixed to prepare alkali-activated material pastes, which limited its further development [15,16,17]. The use of near-neutral salt (i.e., Na_2_CO_3_) as a strong alkali activator substitute not only saves economic costs but also reduces the health and safety risks of processors. However, Na_2_CO_3_ is not conducive to the mechanical performance development of alkali-activated material pastes after hardening [18].

Waste glass is a significant part of municipal solid waste that cannot be ignored. However, it has posed a great challenge to the environment due to its non-degradable properties [19]. Some studies have shown that waste glass contains abundant amorphous silica, which have sufficient pozzolanic activity when ground to a certain fineness [20]. Therefore, it could be used as a new aluminium–silica precursor to partially replace cement [21,22]. However, due to its low pozzolanic reaction, it needs to be activated with a stronger alkaline activator. Ali et al. [23] used a large amount of GP instead of GGBS to prepare alkali-activated materials and found that the incorporation of 75% GP in alkali-activated materials could effectively reduce the severity of efflorescence. Long [24] studied the rheological properties of a GGBS–GP mixture with solid sodium silicate as a single activator and compared it with ordinary Portland cement (OPC) paste, and found that under the same degree of fluidity, the plastic viscosity of alkali-activated GGBS–GP paste was much higher than that of the OPC paste. Shriram [25] used NaOH in collaboration with sodium silicate as a composite activator to activate the ternary mixture of GGBS–FA–GP, and the compressive strength of the matrix showed a tendency to increase and then decrease with the increase in GP content. Cercel J [26] used Na_2_CO_3_ as the major activator and calcium hydroxide as the auxiliary activator to collectively activate a GP–GGBS system, and the results showed that the mechanical properties of the matrix had a more significant improvement compared with using the single-component activator.

CCR is an industrial by-product containing more than 80% calcium hydroxide which could be used as a regenerated calcium source or a green auxiliary alkali activator in alkali-activated materials [27]. Yang et al. [28] used CCR and sodium silicate to synergistically activate FA, and the results showed that the composite activators significantly improved the mechanical properties and hydration products of FA-based geopolymers. Shi et al. [29] prepared an all-solid waste-based geopolymer by using CCR as an alkaline activator and found that CCR could be considered as an effective activator to improve the dissolution of FA and GGBS in alkaline environments, and promote a geopolymer reaction.

However, there have been relatively few studies about GP mixtures activated by CCR-assisted Na_2_CO_3_. Therefore, this paper presents the effect of partial replacement of FA by GP on the rheological and mechanical properties of the ternary mixture system under the synergistic activation of CCR and Na_2_CO_3_. It provides an extremely low carbon footprint route for the preparation of alkali-activated materials and promotes the resource recycling of industrial solid waste materials. In addition, microstructural analysis of the reaction products was carried out utilizing X-ray diffraction (XRD), Fourier transform infrared spectroscopy (FTIR), Thermogravimetric analysis (TGA), and Scanning electron microscopy-Energy dispersive spectrometer (SEM-EDS).

## 2. Materials and Test Methods

### 2.1. Materials

**Figure 1:**
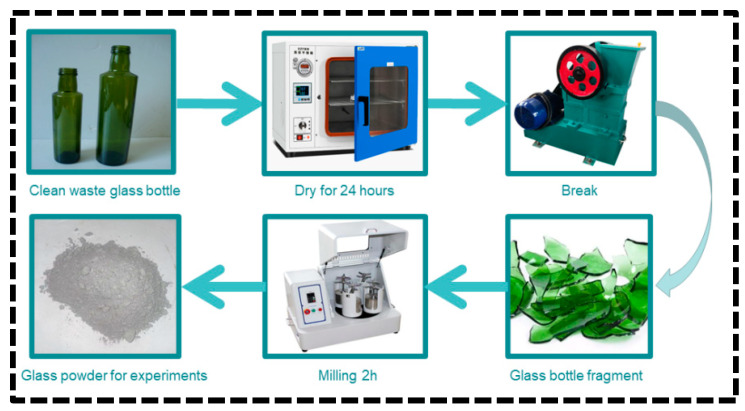
Glass powder preparation process.

**Figure 2:**
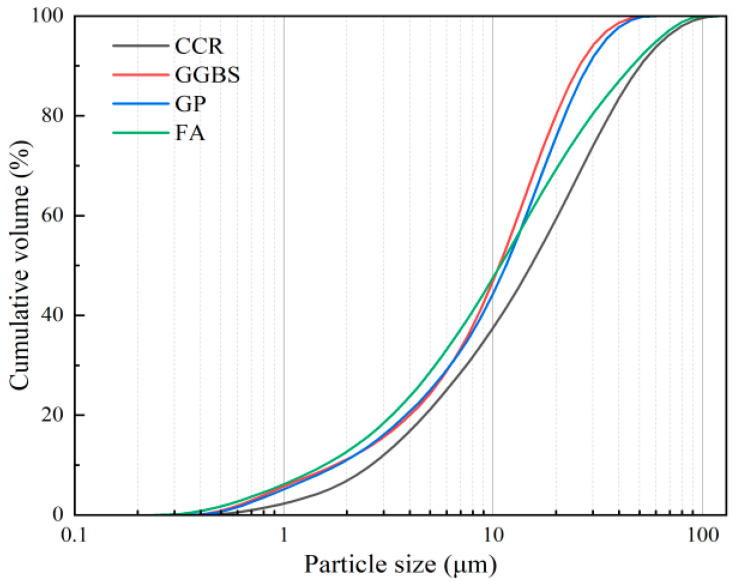
PSD curve of raw materials.

**Figure 3:**
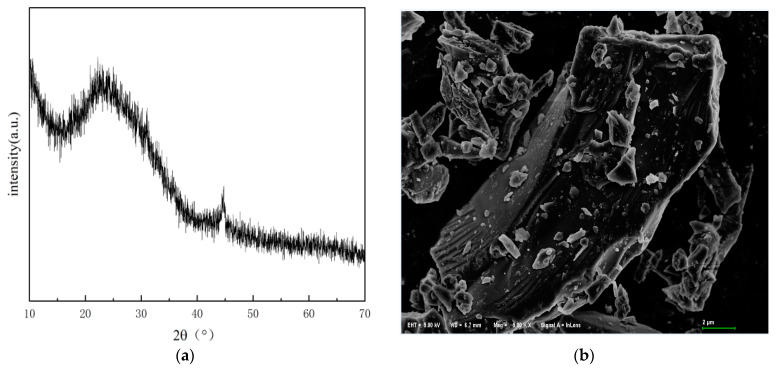
(**a**) XRD of GP; (**b**) SEM of GP.

**Figure 4:**
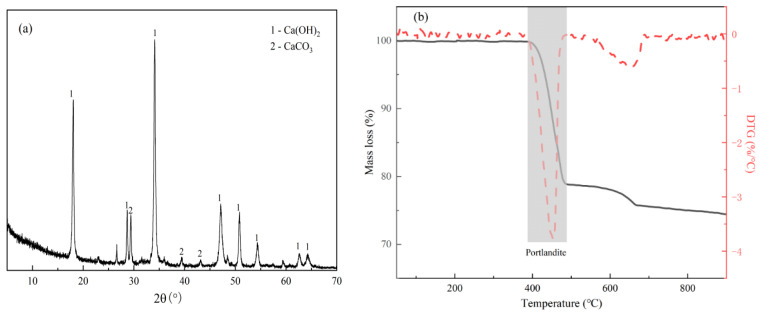
(**a**) XRD of CCR; (**b**) DTG curves of CCR.

In this study, GGBS, FA, and GP were used as precursors for alkali-activated materials. Waste glass bottles collected from a local landfill were cleaned and dried in a vacuum oven at 105 °C for 24 h, then crushed by a small jaw crusher and ground with a small ball mill for 2 h to obtain the GP. The specific process is shown in Figure 1. The microscopic morphology and XRD of the GP are shown in Figure 2. CCR and anhydrous sodium carbonate (Na_2_CO_3_, analytical grade, purity ≥ 98%) were used as composite activators. The chemical composition of various raw materials tested by X-ray fluorescence analysis is shown in Table 1. According to the chemical composition of the GP in Table 1, the waste glass obtained in this paper could be classified as soda silica glass [30]. The composition of soda silica glass is about 65~75% SiO_2_, 12~17% Na_2_O, and 6~12% CaO. The particle size distribution (PSD) of the raw materials is shown in Figure 2. The median size (D50) of the GGBS, FA, GP, and CCR were 10.574 μm, 12.554 μm, 10.850 μm, and 14.532 μm, respectively, and the particle size of the GP was finer than the FA. The physicochemical properties of GP are shown in Table 2 according to ASTM C 618 [31]. Among them, the strength activity index was used to evaluate the pozzolanic activity. The XRD and SEM of GP are shown in Figure 3a,b, respectively. It was observed that the XRD of GP showed diffuse diffraction peaks between 15–30°, which indicated that GP is an amorphous material with pozzolanic activity. From Figure 3b, it can be observed that the surface morphology of GP particles is smooth and irregularly angular, and some of the smaller particles adhere to some larger particles by electrostatic adsorption. The XRD and DTG curves of CCR are shown in Figure 4a,b. The main mineral phase of CCR was observed to be Ca(OH)_2_ from XRD and a little CaCO_3_ was present owing to the carbonation reaction. Based on the mass loss peak between 370–530 °C in the DTG curve of CCR and Equation (1), the content of Ca(OH)_2_ in CCR was 81.5%:

(1) $$CH(\%)=\left[\frac{\left(M_{370}-M_{530})\times (\frac{74}{18}\right)}{M}\right]\times 100\%$$

where *M*_370_ and *M*_530_ are the mass loss of CCR at 370 °C and 530 °C, respectively, and *M* is the total mass of CCR.

### 2.2. The Preparation of CCR Alkali-Activated Composite Cementitious Material

In this paper, CCR and Na_2_CO_3_ were used as composite alkali activators by external doping, and the total amount of doping was 15% of the total mass of the alkali activated material precursors. The CCR alkali-activated materials mixtures with 60% FA and 40% GGBS were used as a reference sample (GP0FA60). Four paste mixtures were prepared by partially replacing FA with GP (named, respectively, GP5FA55, GP10FA50, GP15FA45, and GP20FA40). The mix design of five mixtures is shown in Table 3. To study the different contents of GP replacing FA as the primary variable, the liquid to solid (W/S) ratio was maintained at 0.6 to keep a good workability for all the studied mixtures. In the experiment, the “one-step method” [32] was used to prepare the alkali-activated composite cementitious materials, which could effectively reduce the harmful effects of concentrated alkaline aqueous solutions on the safety of handlers and had certain environmental benefits. The alkali-activated material precursor and the solid base activator were first stirred at a low speed for 2 min to keep the solid mixture homogeneous. Then, water was gradually added and stirred rapidly at 280 rpm for 2 min. Finally, the mixture was mixed slowly at 140 rpm for 1 min. After testing the fluidity and rheological properties, the alkali-activated material paste was put into plastic molds. Specimens were cured under standard conditions (100% RH, 25 °C).

### 2.3. Mini-Slump Flow Diameter

The mini-slump test was used to test the fluidity performance of the alkali-activated materials paste. The mini-slump cone is a conical mold with an open top and bottom, with a top diameter of 38.2 mm, bottom diameter of 57.8 mm, and a height of 57.8 mm. The test was performed according to the EN 1015-3:1999 standard [33]. The fluidity test procedure was to pour the fresh paste of the alkali-activated materials into a small slump cone, then quickly lift the cone and let the paste flow freely on the glass plate for 30 s. The maximum diameter of the paste in two vertical directions was measured with a straightedge, and the average value was taken as the fluidity of the paste.

### 2.4. Test of Rheological Properties

Rheological properties included two parameters of apparent viscosity and shear stress. In this study, after each group of alkali-activated materials paste was stirred with water in the mixer, part of the paste sample was immediately taken for rheological testing under the R/SST 2000 rheometer within 30 s. The specific test procedure was as follows: to ensure that the paste is in a stable state, the rheological test maintained a shear rate of 100 s^−1^ from the beginning of the pre-stress. Then, the shear rate was increased from 0 s^−1^ to 100 s^−1^ within 60 s and then decreased from 100 s^−1^ to 0 s^−1^ within 60 s, and the whole process was repeated twice. The entire rheological test lasted 300 s. The Herschel–Bulkley model was used to fit the rheological data and to estimate the shear stress according to Equation (2), and the equivalent plastic viscosity was calculated according to Equation (3):

(2) $$\tau =\tau_{0}+k\gamma^{n}$$

(3) $$\mu_{p}=\frac{3k}{n+2}\gamma_{max}^{n-1}$$

where *τ* is shear stress (Pa); *τ*_0_ is yield stress (Pa); *k* is coefficient of consistency; *γ* is shear rate (s^−1^); *n* is rheological index, shearing thinning (*n* < 1), and shearing thickening (*n* > 1); and *μ_p_* is the equivalent plastic viscosity.

### 2.5. Test of Mechanical Properties

The fresh alkali-activated materials paste was poured into 40 mm × 40 mm × 40 mm and 40 mm × 40 mm × 160 mm plastic molds, respectively, and covered with plastic film for 1d and then demolded. The alkali-activated materials specimens were cured under standard conditions for 3d, 7d, and 28d, and were tested for compressive strength and flexural strength according to EN 1015-11 [34]. The data of each group of samples were tested three times and the average value was taken.

### 2.6. XRD

The samples cured for 28d were crushed and a small number of fragments were taken and dried in a vacuum drying oven at 45 °C for 24 h, and then ground and passed through a 200-mesh sieve for XRD analysis. XRD was conducted using a D8 Advance X-ray diffractometer, with the tube operated at 40 mA and 40 kV, and a 2θ range of 5–70°.

### 2.7. FTIR

FTIR was performed using a Nicolet 6700 FTIR Spectrometer over the wavelength range of 500 to 4000 cm^−1^ with a resolution of 1 cm^−1^.

### 2.8. DTG

DTG was performed in a STA-449-F3 model integrated with a thermal analyzer. The treated sample powder was put in a small ceramic crucible, and heated from 50 °C to 1000 °C at a rate of 10 °C/min in an environment of nitrogen as a medium, and the flow rate was 50 μL/min.

### 2.9. SEM-EDS

The broken pieces of the sample were taken and dried in a vacuum drying oven for 24 h. Then, the samples were plated with platinum in a vacuum for 480 s and the micro-structure of the broken pieces was observed under a scanning electron microscope. The accelerating voltage of the electron microscope was 0.5–30 kv, and the low vacuum pressure was 1–270 Pa. An X-ray energy spectrometer was used to analyze the composition changes of the samples.

## 3. Results and Discussion

### 3.1. Mini-Slump of the CCR Alkali-Activated Composite Cementitious Materials

**Figure 5:**
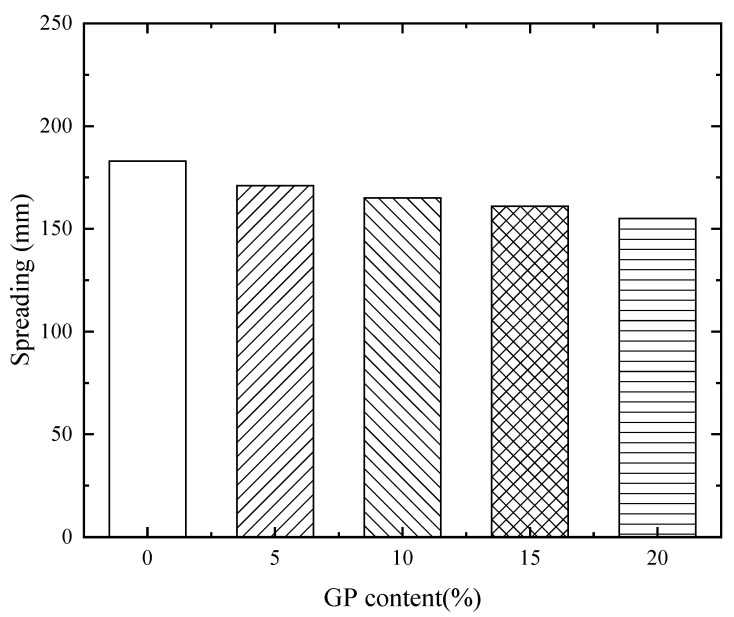
Effect of GP replacing part of FA on the flow of CCR alkali-activated composite cementitious materials paste.

Figure 5 shows the effect of GP replacement for FA under CCR–Na_2_CO_3_ synergistic activation on the fluidity of the alkali-activated materials paste. It can be seen from the Figure that the fluidity of the paste showed a gradual decrease with the increase in GP content. When FA was replaced by GP at levels of 5%, 10%, 15%, and 20%, the fluidity of the paste was decreased by 6%, 9.8%, 12%, and 15.3%, respectively, compared with the reference sample (GP0FA60). On the one hand, this might be attributed to the diamond-shaped morphology of GP particles colliding with each other, which increased the friction during the flowing of the paste. On the other hand, the surface morphology of the GP was more easily disrupted under the synergistic effects of CCR and Na_2_CO_3_ compared to the surface structure of the FA, which caused the increase in the viscosity and decrease in fluidity.

### 3.2. Rheological Behavior of the CCR Alkali-Activated Composite Cementitious Materials

**Figure 6:**
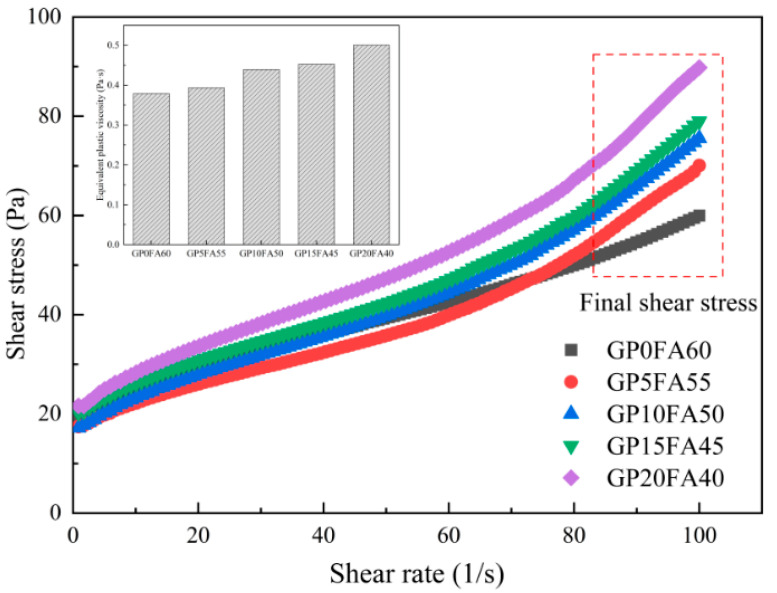
Effect of GP replacing part of FA on the rheological behavior of CCR alkali-activated composite cementitious material paste.

The effect of GP replacing FA on the rheological properties of alkali-activated composite cementitious material paste is shown in Figure 6. The fitting equations based on the Herschel–Bulkley rheological model are summarized in Table 4. It can be observed from the Figure that the shear stress of alkali-activated materials paste showed an increasing trend with the increase in the shear rate. When the shear rate reached the maximum critical value, the shear stress of the alkali-activated materials with 5%, 10%, 15%, and 20% FA replaced by GP was 70 Pa, 75.5 Pa, 78.9 Pa, and 89.8 Pa, respectively, which increased by 16.9%, 25.8%, 31.7%, and 49.6%, indicating that at the same shear rate, the shear stress of alkali-activated materials paste increased with the increase in GP. This could be explained by the interparticle friction effect of the different morphologies of the solid precursors, where the irregular morphology of GP increased the interparticle friction in the paste compared to the spherical morphology of FA [35]. In addition, the pastes provided different trends at a low shear rate as compared to a high shear rate. This might be explained that at low shear rates, the less doped GP could not sufficiently react with the alkaline activator, which resulted in less flocculent products and relatively low shear stress. With the increase in shear rate, the opportunity of contact between the GP and alkaline activator was increased, the reaction was comparatively more adequate, more flocculent products were produced, and the shear stress was increased [36].

It can also be observed from the Figure that the equivalent plastic viscosity of the alkali-activated materials paste showed a similar increasing trend with the increase in FA replacement by GP. When FA replaced by GP was 5%, 10%, 15%, and 20%, the equivalent plastic viscosity of the alkali-activated materials paste was 0.39 Pa·s, 0.43 Pa·s, 0.45 Pa·s, and 0.5 Pa·s, respectively, which were respectively increased by 3.9%, 15.9%, 19.5% and, 32.1%, compared to the reference sample (GP0FA60). It is well known that the equivalent plastic viscosity is an important index to measure the difficulty of paste flow, which is closely related to fluidity. As FA was gradually replaced by GP, the viscosity of the alkali-activated material paste was increased and the fluidity was decreased, which is consistent with the results in Section 3.1. The reason for this phenomenon is that the irregular diamond shape of GP leads to increased interaction forces between particles compared to the spherical shape of FA, resulting in difficult paste flow [37]. On the other hand, under the synergistic effects of CCR–Na_2_CO_3_, the surface structure of GP with a finer particle size was more easily destroyed than FA, which increased the shear stress and equivalent plastic viscosity of alkali-activated materials paste [38].

**Figure 7:**
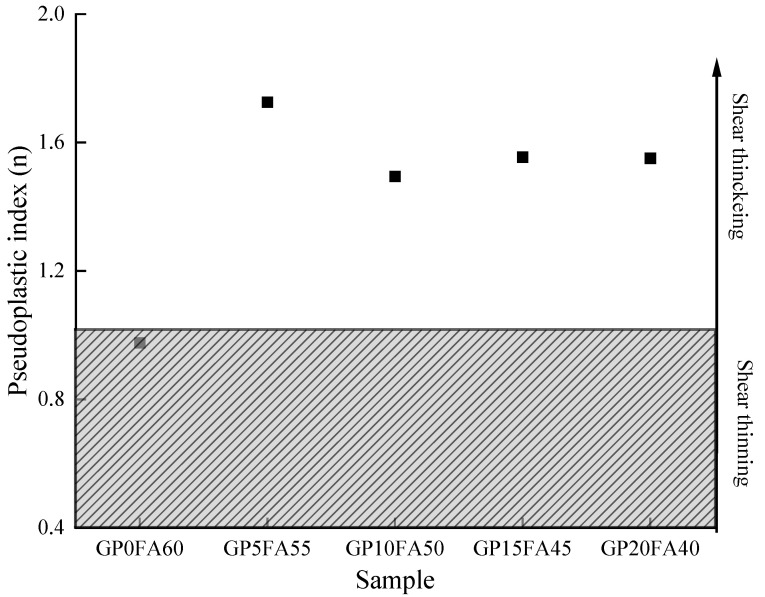
Variation of pseudoplasticity index (*n*) with GP replacing FA in the CCR alkali-activated composite cementitious material paste.

In addition, it can be seen from Figure 7 that when the shear rate increased, the reference sample showed an apparent shear thinning phenomenon (*n* < 1) and the pseudoplastic index (*n*) was 0.97. This was attributed to the relatively slow reaction rate of the alkali-activated materials matrix under the synergistic activation of CCR and Na_2_CO_3_, and the spherical shape of FA had a “ball bearing” effect, which reduced the plastic viscosity of the paste [39]. With FA gradually replaced by GP, the alkali-activated materials paste changed from a shear-thinning state to a shear-thickening state (*n* > 1) under high-speed shearing. When FA was replaced by 5% GP, the pseudoplasticity index (*n*) was 1.72 and apparent shear thickening was observed, which could be attributed to the fact that the incorporation of GP accelerated the reaction rate of the alkali-activated materials matrix under the synergistic activation of CCR and Na_2_CO_3_. The pseudoplasticity index decreased slowly and showed a fluctuating trend with the increase in GP, which might be related to the degree of reaction between CCR and Na_2_CO_3_. The concentration of sodium hydroxide produced by the reaction of CCR and Na_2_CO_3_ in a short period was limited, which was not enough to activate more GP [40].

### 3.3. Mechanical Properties of the CCR Alkali-Activated Composite Cementitious Materials

**Figure 8:**
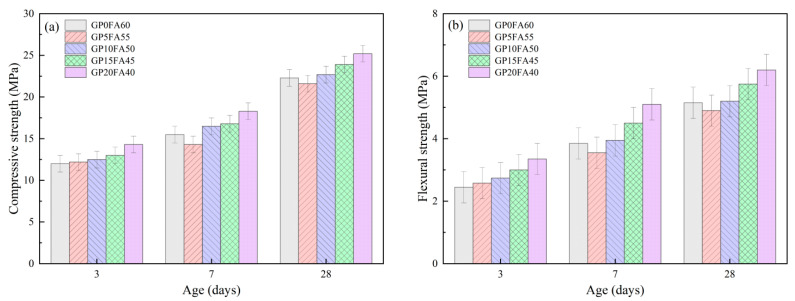
Effect of GP replacing part of FA on the mechanical properties of CCR alkali-activated composite cementitious materials, (**a**) Compressive strength; (**b**) Flexural strength.

Figure 8a,b shows the effect of partial replacement of FA by GP on the compressive and flexural strength of the alkali-activated material matrix under the synergistic activation of CCR and Na_2_CO_3_. It can be seen that the compressive and flexural strengths of the alkali-activated materials matrix both gradually increased with the increase in GP when the curing period was 3d. When FA was replaced by 20% GP, its compressive strength and flexural strength increased by 16.2% and 36.7%, respectively, compared to the reference sample (GP0FA60). This might be attributed to the higher reactivity of GP at an early age, and the reaction of CCR and Na_2_CO_3_ to generate NaOH and CaCO_3_; thus, more soluble reactive silicon was produced under the activation of NaOH (as shown in chemical reaction Equations (4) and (5)). When the curing time was increased to 7d and 28d, the compressive and flexural strengths of the alkali-activated materials matrix both showed a tendency to decrease and then increase with the increase in GP replacement for FA, which is similar to the research results of Khan [41]. This might be attributed to the diamond-shaped microscopic morphology of GP compared to FA, which destroyed the original stacked skeletal structure of the alkali-activated materials matrix and thus harmed the development of the matrix strength of the alkali-activated materials. However, when GP replacement for FA exceeded 10%, the factors controlling the growth of alkali-activated materials matrix strength changed from physical to chemical factors, and it was found that the compressive strength of the GP15FA45 and GP20FA40 samples was increased by 7.17% and 13%, respectively, and the flexural strength was increased by 11.6% and 20.3%, respectively, compared with the reference samples (GP0FA60) at 28d. The alkali-activated materials using CCR and Na_2_CO_3_ synergistically activated showed better mechanical properties than Samarakoon [32] using sodium hydroxide as a single alkali activator. This was attributed to the fact that CaCO_3_ produced by the reaction of CCR with Na_2_CO_3_ had a fine crystalline structure, which could fill the pores and improve the strength of the matrix [42].

(4) $$Na_{2}CO_{3}+Ca{\left(OH\right)}_{2}\to 2NaOH+CaCO_{3}$$

(5) $$6NaOH+2H_{2}O+4CaCO_{3}\to 3Ca{\left(OH\right)}_{2}+Na_{2}Ca(CO_{3})\cdot 2H_{2}O+2Na_{2}CO_{3}$$

### 3.4. XRD Analysis

**Figure 9:**
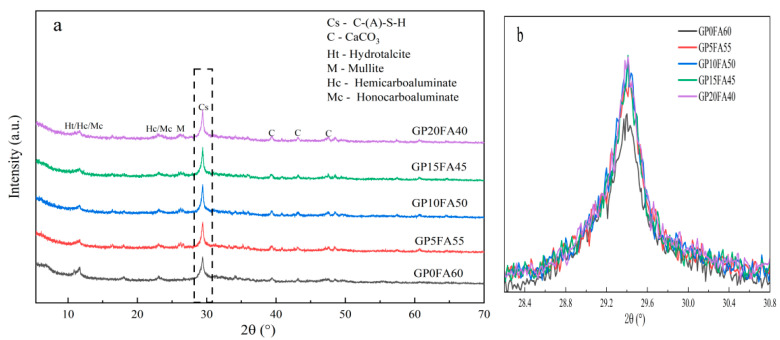
(**a**) The XRD of the CCR alkali-activated composite cementitious material at 28d; (**b**) the XRD local magnification of the hydrated product with a diffraction angle of 29.2°.

Figure 9a shows the XRD results, at 28d, of the alkali-activated materials with GP replacing FA, and Figure 9b shows the XRD local magnification of the hydrated product with a diffraction angle of 29.2°. From Figure 9a, it can be seen that the partial replacement of FA by GP under the synergistic activation of CCR and Na_2_CO_3_ produced the following products: C-(A)-S-H, hydrotalcite, calcium hemicarboaluminate (Hc), calcium monocarboaluminate (Mc), calcium carbonate, and mullite. The main crystalline phase C-(A)-S-H gel was located at 29.2°. Some of the Al^3+^ in the raw material could enter the C-S-H gel to replace some of the Si^4+^ in the crystal structure to form C-(A)-S-H gel, which increased the degree of polymerization of silica tetrahedra, leading to longer silica chain lengths and an increased C-(A)-S-H gel interlayer distance [43,44,45]. In addition, it can be seen from Figure 9b that the intensity of the diffraction peak of C-(A)-S-H gradually increased with the increase in GP replacement for FA, indicating that under the synergistic activation of CCR and Na_2_CO_3_, GP had some effect on the mineral phase composition of the matrix of the alkali-activated material. The peak of hydrotalcite was located at 11.2°, similar to the research results of Zhang [46]. This was attributed to the high alkaline environment (PH > 9) formed by the synergistic activation of CCR and Na_2_CO_3_, which promoted the deposition and crystallization of Mg^2+^, Al^3+^, and CO_3_^2−^ in the pore solution from GP and GGBS.

### 3.5. FTIR Analysis

**Figure 10:**
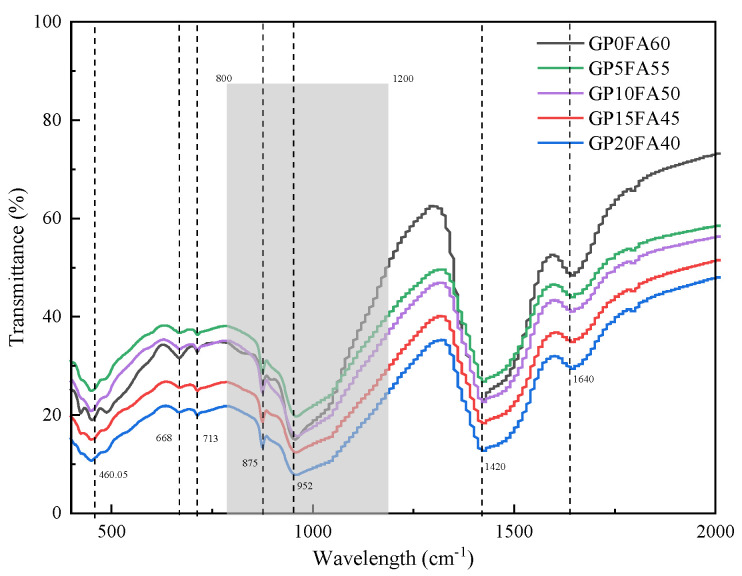
The FTIR graph of CCR alkali-activated composite cementitious material at 28d.

To further understand the results of the XRD analysis, Figure 10 shows the effect of GP replacement for FA on the FTIR of the matrix of the alkali-activated materials at 28d. Many studies have shown the peaks at wavelengths between about 800 cm^−1^ and 1200 cm^−1^ (the shadowed region), which are the overlapping peaks between precursors, and that reaction products could be used as a focus for studying the crystal structure changes of non-crystalline C-(A)-S-H gels [47,48]. Under the synergistic activation of CCR and Na_2_CO_3_, the peak of the amorphous C-(A)-S-H gel in the matrix was concentrated at 952 cm^−1^, which was the result of asymmetric stretching and oscillation of the Si-O bond or the Si-O-Si bond (alkali metal or alkaline earth metal) [49]. In the early stage of alkali activation, the bond energy of the Al-O bond was low relative to that of the Si-O bond, which was more likely to dissolve in the pore solution in the alkali-activated matrix and replace Si in the silicon-oxygen tetrahedra to form the Al-rich phase C-(A)-S-H. As the hydration age increased to 28d, the position of the peak moved towards higher wavelengths, and a highly alkaline environment was formed by the synergistic activation of CCR and Na_2_CO_3_ to promote the dissolution of GP. It led to more soluble silicon in the amorphous C-(A)-S-H gel structure to form a silica-rich phase, increased the polymerization of the C-(A)-S-H gel, and formed a more stabilized gel.

### 3.6. DTG Analysis

**Figure 11:**
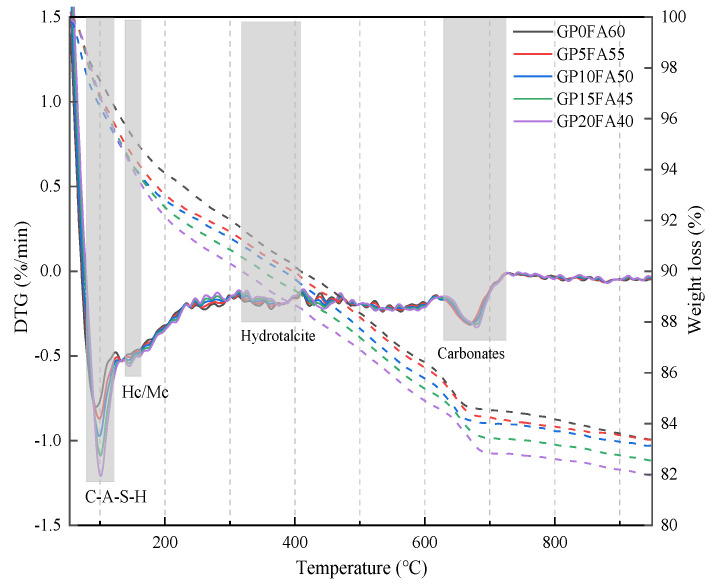
DTG curves of CCR alkali-activated composite cementitious material at 28d.

Figure 11 shows the DTG curves of the alkali-activated materials matrix under the synergistic activation of CCR–Na_2_CO_3_ with GP replacing FA. The observed heat absorption peak at 80–150 °C in the Figure was mainly attributed to the dehydration of the C-(A)-S-H gel phase; such results are in agreement with the study of Xiao [50]. With the increase in GP replacement for FA, the intensity of the heat absorption peak gradually increased in the range of 80–150 °C, indicating that more C-(A)-S-H gels were generated in the alkali-activated materials matrix, which was consistent with the results in XRD. The very weak loss peaks were observed at the shoulder in the range of 150 °C to 210 °C and the loss peaks were observed in the range of 300 to 400 °C, mainly related to the dehydration decomposition of Hc/Mc and Ht, respectively. The heat absorption peak present at 600 °C to 750 °C and the weak peak after 750 °C in the Figure were attributed to the decomposition of the CO_3_^2−^ containing phase [41,46,51].

**Figure 12:**
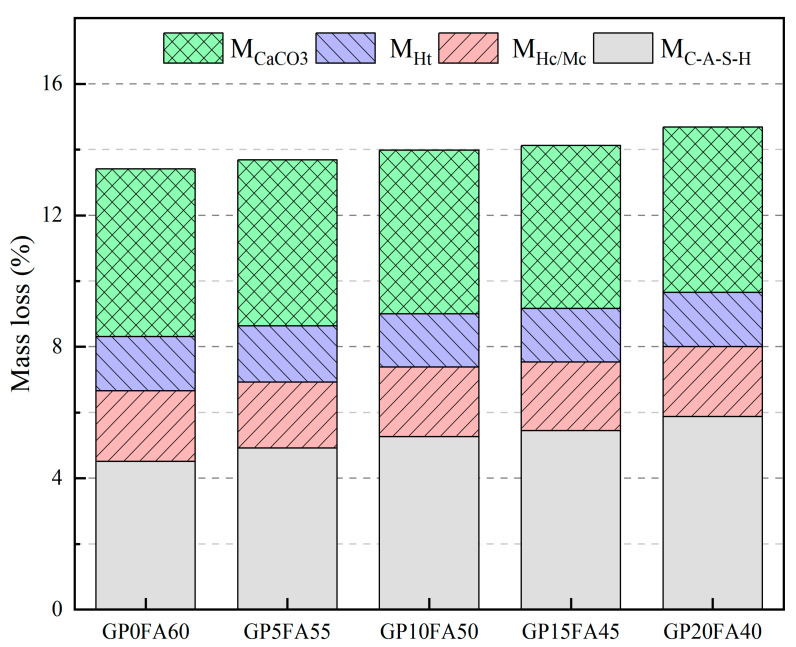
Mass loss of C-A-S-H gel, Hc/Mc, hydrotalcite and calcite phase.

The mass loss of each hydration product corresponding to the temperature range is shown in Figure 12. It can be observed from the Figure that as GP replacement for FA increased from 0% to 20%, the total mass loss was increased from 15.8% to 17.4%, and the mass loss of C-A-S-H gel was increased from 4.5% to 5.9%, while the mass loss of other hydration products remained almost constant. This indicated that the effect of GP on the hydration process was mainly reflected in the dehydration decomposition of C-(A)-S-H gels, and GP could promote the gel generation, which is conducive to the development of matrix strength.

### 3.7. SEM/EDX Analysis

**Figure 13:**
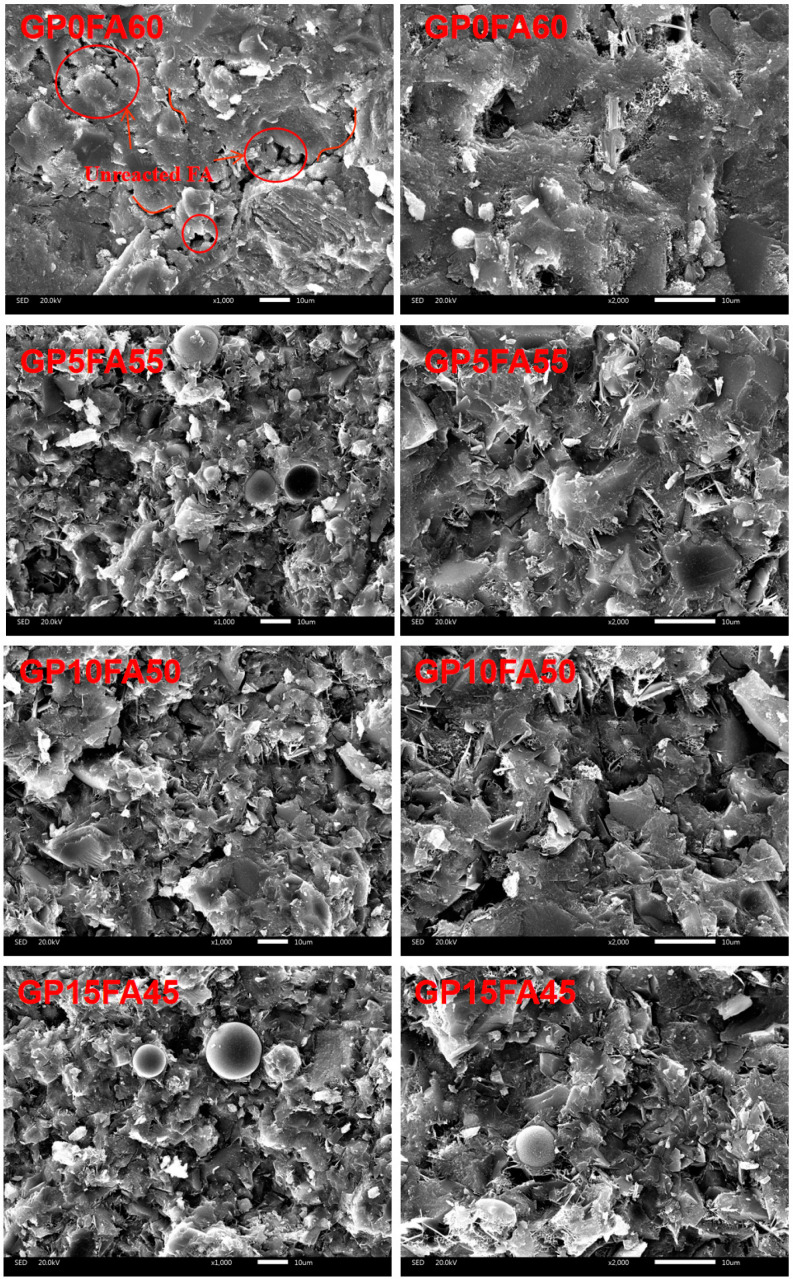

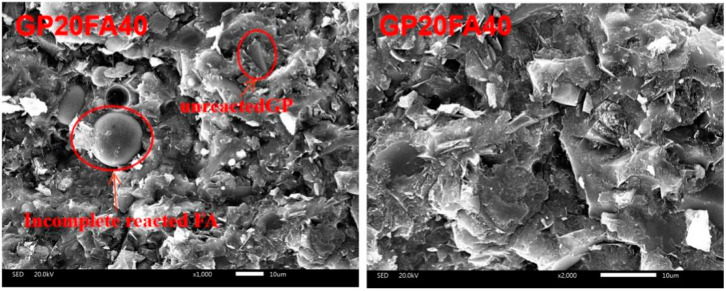
SEM of CCR alkali-activated composite cementitious material at 28d.

Figure 13 shows the microstructure of alkali-activated composite material at 28d under the synergistic activation of CCR and Na_2_CO_3._ It can be observed that the more incompletely reacted FA particles were inserted inside the matrix in the sample GP0FA60. Furthermore, many cracks and pores were observed in the sample GP0FA60, which were detrimental to the strength of the matrix. The SEM of sample GP20FA40 showed that no apparent cracks existed on the surface structure of the matrix, but some incompletely reacted FA was present. The incorporation of GP might consume the concentration of the alkali activation solution, resulting in an insufficient concentration of the alkali activation solution required for FA, which inhibited the destruction vitreous structure of FA and generated relatively more hydration products in the neighborhood of the GP particles [51]. In addition, the incompletely reacted FA was able to fill the pores of the matrix as an inert aggregate, which improved the mechanical properties to some extent [52].

**Figure 14:**
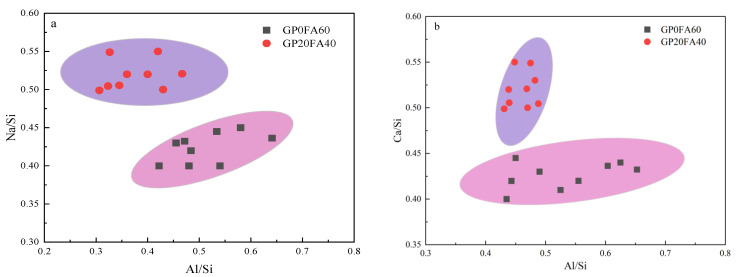
Microstructure of alkali-activated materials based on SEM-EDS, (**a**) The relationship between Na/Si and Al/Si; (**b**) Relationship between Ca/Si and Al/ Si.

Figure 14a,b further compares the effect of GP on Ca/Si, Na/Si and Al/Si in the matrix of the alkali-activated materials. It can be seen from the Figure that the Al/Si ratio of GP0FA60 ranged from 0.42 to 0.66, while the Al/Si of GP20FA40 ranged from 0.3 to 0.48. This can be attributed to the reduction in the Al content in the matrix due to the partial replacement of FA by GP, which further decreased the Al/Si content in the C-A-S-H gel. In addition, it can be seen from Figure 14a that the Na/Si ratio of GP20FA40 was higher than GP0FA60, which was attributed to the fact that the surface of the GP was more susceptible to being destroyed than that of the FA under the highly alkaline environment formed by the reaction of CCR and Na_2_CO_3_. From Figure 14b, it can be seen that with the increase in GP content, the Ca/Si ratio of the gel gradually increased, the distribution became more concentrated, and the degree of polymerization increased, which had a compensating effect on the negative charge. In addition, GP with higher CaO content had a higher space-filling ability and promoted the formation of additional C-(A)-S-H gels compare to FA, which could contribute to the improvement of the microstructure and mechanical properties of the matrix.

## 4. Conclusions

This paper investigated the feasibility of waste glass as a precursor in the carbide residue composite cementitious material system. The rheological behavior, mechanical properties, and microstructure of carbide residue alkali-activated materials were analyzed through a series of tests. The following conclusions were derived from the results of the study:

1. The substitution of GP for FA had a negative effect on the fluidity of the alkali-activated materials paste under the synergistic activation of CCR and Na_2_CO_3_, which was attributed to the diamond-shaped morphological structure of GP that increased the friction between particles during the flow of the alkali-activated materials paste.
2. The rheological behavior of alkali-activated materials incorporating GP conformed to the Herschel–Bulkley model. Compared to the reference, when the partial replacement of FA by GP was increased to 20%, the shear stress was increased by 49.6% and the equivalent plastic viscosity increased by 32.1%.
3. With the increase in FA replaced by GP, the alkali-activated materials paste gradually changed from a shear thinning to shear thickening response. When 5% GP was used as a replacement for FA, the maximum pseudoplasticity index was 1.72.
4. When 20% GP was substituted for FA, the compressive strength and flexural strength of alkali-activated materials at 28d were increased by 13% and 20.3%, respectively, compared with the reference sample.
5. Under the co-activation of CCR and Na_2_CO_3_, GP replacing part of FA contributed to the dissolution of Ca and Si to generate C-(A)-S-H gels with higher polymerization, which promoted the development of the mechanical properties of the substrate.

**Recommend:** The present study was limited to investigating the effect of glass powder as a cementitious material on the performance of alkali-activated materials, and it is recommended to further explore the synergistic effect of glass powder as a cementitious material in conjunction with glass sand as a fine aggregate in alkali-activated materials. 

## Figures and Tables

**Table 1:** Chemical composition of raw materials (%).

Raw Materials	Na_2_O	MgO	Al_2_O_3_	SiO_2_	P_2_O_5_	SO_3_	K_2_O	CaO	Fe_2_O_3_
GGBS	0.46	7.41	14.91	30.39	0.03	2.22	0.42	42.41	0.44
FA	0.33	0.23	38.01	46.44	0.06	0.69	0.88	7.52	3.12
GP	12.76	1.97	2.24	68.48	0.02	0.23	0.33	10.81	0.95
CCR	0.02	0.09	1.38	2.72	0.01	0.77	0.04	77.99	0.42

**Table 2:** The physical and chemical properties of GP.

Testing Items		Limits
	WGP	Class N
SiO_2_ + Al_2_O_3_ + Fe_2_O_3_	71.67%	>70%
SO_3_	0.23%	<4%
Moisture content	1.48%	<10%
Loss on ignition	2.21%	<3%
Amount retained when wet-sieved on 45 μm sieve	11.4%	<34%
Strength activity index at 7 days	81.2%	>75%
Strength activity index at 28 days	85.3%	>75%

**Table 3:** Design composition of CCR alkali-activated composite cementitious material.

Sample	GP	FA	GGBS	CCR	Na_2_CO_3_	W/S
GP0FA60	0	60%	40%	6%	9%	0.6
GP5FA55	5%	55%	40%	6%	9%	0.6
GP10FA50	10%	50%	40%	6%	9%	0.6
GP15FA45	15%	45%	40%	6%	9%	0.6
GP20FA40	20%	40%	40%	6%	9%	0.6

**Table 4:** The fitting equation of the alkali-activated materials paste.

Sample	The Fitting Equation	R^2^	*n*
GP0FA60	y = 20.42 + 0.419x^0.9765^	0.9915	0.9765
GP5FA55	y = 18.79 + 0.01626x^1.726^	0.9908	1.726
GP10FA50	y = 21.77 + 0.05239x^1.495^	0.9945	1.495
GP15FA45	y = 22.46 + 0.03984x^1.555^	0.9926	1.555
GP20FA40	y = 26.97 + 0.04683x^1.551^	0.9937	1.551

## Data Availability

The data presented in this study are available on request from the co-responding author.
